# Cellular diversity in neural organoids is critical for neurodegenerative disease modeling

**DOI:** 10.1371/journal.pbio.3004074

**Published:** 2026-10-14

**Authors:** Shiying Liu, Shi-Yan Ng

**Affiliations:** 1 Genome Institute of Singapore (GIS), Agency for Science, Technology and Research (A*STAR), Singapore, Singapore; 2 Institute of Molecular and Cell Biology (IMCB), Agency for Science, Technology and Research (A*STAR), Singapore, Singapore; School of Medicine and Health, Technical University, Munich, GERMANY

## Abstract

Neuroinflammation and vascular dysfunction are hallmarks of most neurodegenerative diseases. This Perspective argues that immune and vascular cells need to be fundamental components of organoids intended to model neurodegeneration, rather than optional extras.

## Teaser

Neuroinflammation and vascular dysfunction are hallmarks of most neurodegenerative diseases. For organoids intended to model neurodegeneration, immune, and vascular cells should therefore be considered fundamental components, rather than optional additions.

Human induced pluripotent stem cell (iPSC)-derived organoids have transformed our ability to model human brain development and neurodevelopmental disorders. Their value lies precisely in their ability to reconstruct cellular diversity and three-dimensional structures and interactions that cannot be captured in traditional two-dimensional cultures. Yet, when these same technologies are applied to neurodegenerative diseases, a familiar reductionist paradigm emerges: introduce a disease-associated mutation, generate neurons within organoids, and develop assays to probe neuronal functional and survival. While this approach is still essential for advancing our understanding of intrinsic defects in neurons, such as impaired autophagy, defective metabolism, loss of protein homeostasis, and changes in electrophysiology, this does not address the noncell-autonomous contributions of neurodegeneration. Unlike many neurodevelopmental disorders, neurodegeneration is not driven solely by intrinsic defects in neural stem cells or early neuronal development, but emerges from both intrinsic neuronal defects as well as changes in dynamic interactions between neurons, glia, vascular cells, immune cells, and their microenvironment over decades of aging. Therefore, we believe that organoids lacking immune or vascular components miss the fundamental biology of neurodegeneration.

In neurodegeneration, although specific subtypes of neurons are the cells that will ultimately die, neuronal death is unlikely to represent the beginning of the disease process. Increasing evidence indicates that astrocytes [1,2], oligodendrocytes [3,4], microglia [1,5], and endothelial cells [6,7] undergo disease-associated changes before overt neurodegeneration. Neurodegeneration is therefore increasingly understood as the consequence of both cell-autonomous and noncell-autonomous processes, in which pathological changes in one cell type can initiate or amplify dysfunction in another. This broader view may also help explain why therapeutic strategies focused primarily on neuronal protection have yielded limited clinical success despite decades of intensive research, and highlights the need to consider nonneuronal cells and pathological cell–cell interactions as therapeutic targets [8]. If the disease is initiated or sustained by interactions between multiple cell types, protecting the neurons alone may be insufficient.

The next frontier in neurodegenerative disease modeling should therefore not simply be to make brain organoids more complex, but to make them more relevant to the disease context. If neuroinflammation is a fundamental component of pathogenesis, then immune cells must be incorporated into the organoids. If vascular dysfunction is central to disease pathology, then endothelial cells should be incorporated into the organoids [9]. The objective should not be to recreate every component of the human brain, but rather to reconstruct the cellular interactions that are necessary to model the disease process.

Among the nonneuronal cells implicated in neurodegeneration, microglia deserve particular attention. Microglia are the resident immune cells of the central nervous system that respond to changes in the extracellular environment by adopting a continuum of phenotypes ranging from pro-inflammatory to anti-inflammatory. Microglia are highly heterogeneous, exhibiting diverse molecular and functional states that emerge in response to disease, injury, or changes in the local microenvironment [10]. For many years, microglia were regarded primarily as responders to neuronal injury [11]. Activated microglia were considered a consequence of neurodegeneration rather than primary contributors. This view has changed substantially. The genetics of neurodegenerative disease provide compelling evidence for a causal role of microglia. For example, mutations in *TREM2* were first identified in 2012 as genetic risk factors for Alzheimer’s disease (AD) [12]. Within the brain, TREM2 is primarily expressed in microglia and functions as a cell surface receptor for sensing the cellular damage that drives phagocytosis. Studies have now convincingly shown that replacing Trem2-mutant microglia with Trem2 wild-type microglia reduces disease phenotypes in a mouse model of AD [13]. These observations suggest that microglial dysfunction influences the initiation and progression of neuronal disease phenotypes. If microglial dysfunction can influence neurodegenerative disease progression, then an organoid that excludes microglia would be unable to replicate a key cellular process that it is intended to model.

These findings have direct implications for therapeutic discovery. Most phenotypic screening in neurodegeneration centers on neuronal mono-cultures, with compounds evaluated according to relatively simple endpoints such as neuronal survival, neurite morphology, or protein aggregation. These assays are amenable to high-throughput screening but assume that the therapeutic target resides within the neuron. But what if it does not? A drug candidate that protects neurons in isolation may fail in vivo because it does not correct the pathological neural microenvironment. If it is true that neurodegenerative diseases such as AD and amyotrophic lateral sclerosis (also known as motor neuron disease) are, at least in part, driven by early dysfunctions of nonneuronal cells such as microglia, then therapeutic screening paradigms based primarily on neuronal mono-cultures may require fundamental re-evaluation (Fig 1).

**Fig 1:**
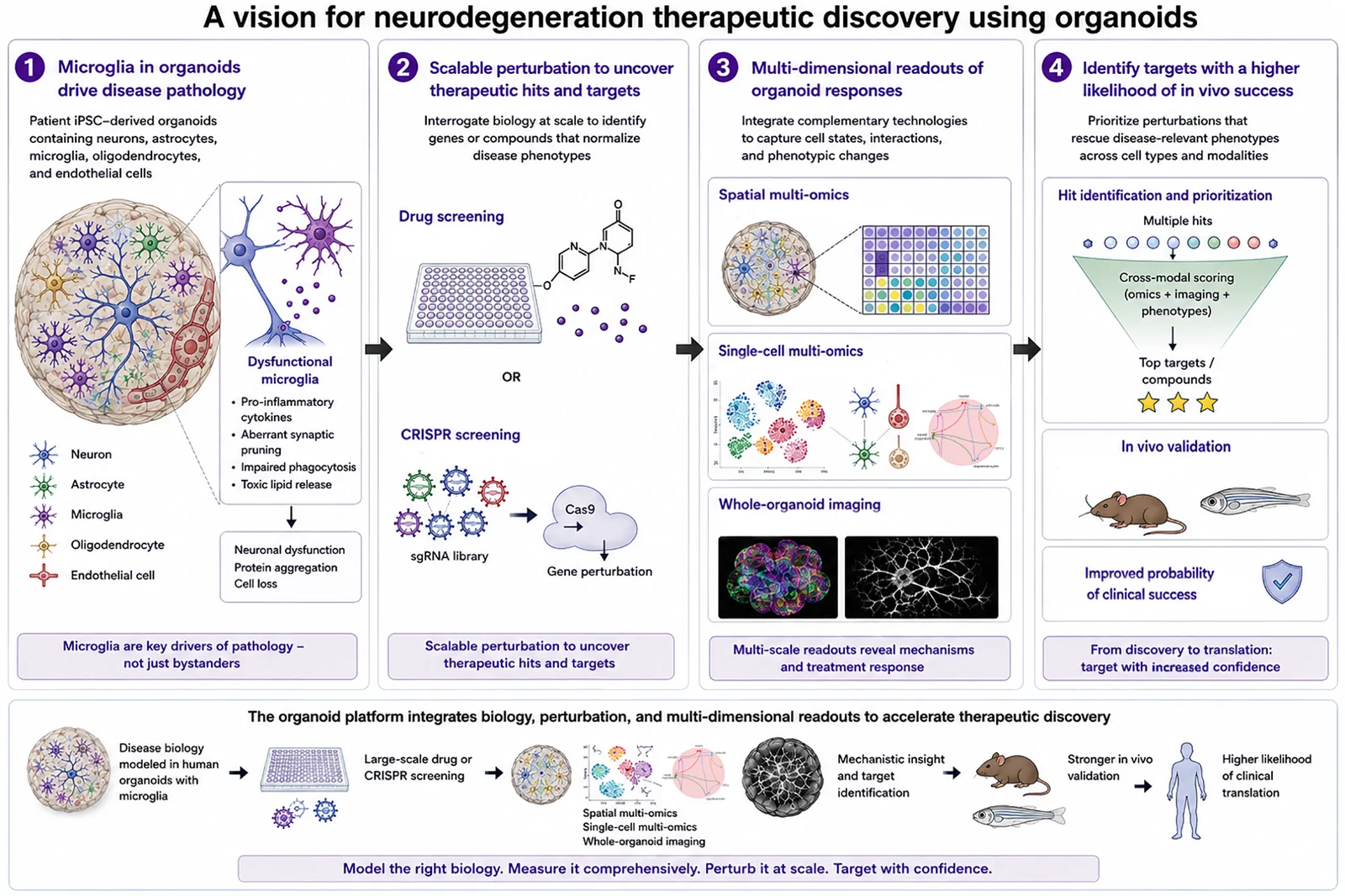
A vision for organoid-based therapeutic discovery in neurodegeneration. Human induced pluripotent stem cell (iPSC)-derived organoids incorporating microglia and other disease-relevant cell types provide a good approximation of neuroimmune crosstalk in neurodegeneration. Scalable small molecule or CRISPR perturbation screens are coupled with spatial and single-cell multi-omics and whole-organoid imaging to identify perturbations that reverse disease-associated phenotypes and uncover therapeutic targets. Integrative analysis enables prioritization of targets for in vivo validation, with the goal of improving the likelihood of therapeutic success. Figure generated with the help of ChatGPT (OpenAI, GPT-4o).

To narrow the gap between neurodegenerative disease research and downstream clinical translation, the field should move towards explicit benchmarks for organoids designed to model neurodegeneration. At a minimum, organoids intended to model neurodegenerative diseases should contain not only relevant neuronal and astrocytic populations, but also a functional neuroimmune component, including microglia. Importantly, the mere presence of microglia should not be considered sufficient. These cells should exhibit appropriate maturation, spatial localization, and molecular states in response to the neural environment. Where vascular dysfunction is central to a particular disease, vascular components should similarly be incorporated. Ultimately, the benchmark should be disease relevance, rather than cellular complexity for its own sake. No disease model can fully recapitulate the complexity of human disease, but what matters is to clearly define the biological questions the model is designed to address, as well as the limitations as to what it cannot capture.

As organoid systems become increasingly complex, technologies that can deconvolute multicellular interactions will become equally important. Single-cell and spatial profiling can determine whether the cellular states within organoids resemble those observed in human disease, while genetic and pharmacological perturbation can establish whether specific cell types or signaling pathways are causal rather than merely correlated with pathology. Whole-organoid imaging, automated phenotypic screening and computational modeling can further enable longitudinal interrogation of how disease-associated states emerge, interact, and propagate across the organoid (Fig 1).

Ultimately, the value of next-generation organoids will not be determined by how closely they resemble the human brain anatomically, but by whether they enable the reconstruction of the cellular interactions that drive disease. For neurodegeneration, we believe microglia are central to this equation. An organoid without microglia might still be a useful model, but for diseases in which neuro-immune dysfunction is integral to pathogenesis, it should no longer be considered a complete model of neurodegeneration.
